# Individual, household and community level factors associated with keeping tuberculosis status secret in Ghana

**DOI:** 10.1186/s12889-016-3842-y

**Published:** 2016-11-25

**Authors:** Joshua Amo-Adjei

**Affiliations:** 1 African Population and Health Research Center, Nairobi, Kenya; 2 Department of Population and Health, University of Cape Coast, Cape Coast, Ghana

**Keywords:** Tuberculosis status, Secret, Disclosure, Ghana

## Abstract

**Background:**

In tuberculosis (TB) control, early disclosure is recommended for the purposes of treatment as well as a means of reducing or preventing person-to-person transmission of the bacteria. However, disclosure maybe avoided as a means of escaping stigma, and possible discrimination. This study aimed at providing insights into factors associated with intentions of Ghanaians to keep positive TB diagnosis in their families’ a secret.

**Methods:**

The paper was based on data from the 2014 Ghana Demographic and Health Survey. Descriptive statistics of proportions with Chi-square test and binary logistic regression were used to identify individual, household and community level factors that predicted the outcome variable (keeping TB secret).

**Results:**

Women were more inclined (33%) than men (25%) to keep TB in the family a secret. Views about keeping TB secret declined with age for both sexes. For women, higher education had a positive association with whether TB in the family would be kept a secret or not but the same was not observed for men. In a multivariable regression model, the strongest predictor of keeping TB secret was whether the respondent would keep HIV secret, and this was uniform among women (OR = 6.992, *p* < 0.001) and men (OR = 9.870, *p* < 0.001).

**Conclusion:**

Unwillingness towards disclosing TB status in Ghana is associated with varied socioeconomic and demographic characteristics, which may be driven by fears of stigma and discrimination. Addressing TB-related stigma and discrimination can enhance positive attitudes towards TB disclosure. For an infectious disease such as TB, openness towards status disclosure is important for public health.

## Background

Cure for tuberculosis (TB) has been in existence for the past 70 years leading to significant progress in reducing the global burden of the disease. Yet, the expected decline has not been very impressive, with annual incidence decline of about 2% with about 9 million cases per annum [1, 2]. Of the estimated cases, about 3 million are missed to treatment, leading to the persistence of infectious cases and further transmission [2]. These gaps have been linked to the fact that a high proportion of those with the disease are not diagnosed and treated. This calls for finding those infected and treated immediately to render the disease non-infectious, thereby, cutting new infections. As CM Yuen, F Amanullah, A Dharmadhikari, EA Nardell, JA Seddon, I Vasilyeva, Y Zhao, S Keshavjee and MC Becerra [3] observe, “if the global TB epidemic is to be stopped, not only must existing cases be treated, but the transmission that is constantly producing new infections and cases must also be halted” (p. 1). Thus, for those infected and their households, early disclosure is very critical in order to facilitate receiving healthcare as well as giving persons in the range of possible transmission an opportunity to take precautionary measures against infection in non-judgemental and non-discriminatory ways.

Historical evidence shows that TB patients were demonized and isolated due to fear of their infectivity [4]. The development of therapeutic and inexpensive chemotherapy in the 1940s ominously minimized the stigma and indignities associated with TB due to fear of infection and death [5]. However, the relationship between human-immuno deficiency virus (HIV) and TB is rekindling and increasing suspicions and labelling of TB patients as persons living acquired immune deficiency syndrome (AIDS) [6–8]. This is reviving stigma towards TB [7].

In Goffman’s [9] classic study on stigma, he emphasised that stigma occurs through discrediting individuals who are negatively regarded by the broader society and are devalued, shunned with lessened life chances. In respect of TB, stigma can lead to unwillingness of individuals to disclose their status which arises from fear of loss of economic opportunities [10], loss of community respect [11], fear of transmission, shame, blame and judgement [12] and questions of “how did you or did she/he get it” [13] and even death.

However, evidence shows that when TB status is disclosed early to ‘significant others’ (e.g. health workers, relatives), infected persons are often supported and encouraged to comply with treatment [14]. In a study by D Zolowere, K Manda, B Panulo Jr, A Muula, DZKMB Panulo and JA Muula [14], the authors demonstrated that persons infected with TB were willing to disclose their status if they felt they would not be stigmatized and that disclosure was facilitated by trust, a feeling of safety, and a sense of obligation.

TB control in Ghana in the last 20 years has recorded a number of successes, pushing treatment success from 40% in the 1990s to around 86% in 2014. These successes have been achieved through a number of interventions such as the enablers’ support,^1^ free medical examination and treatment coupled with improving human and infrastructural resources [15]. Notwithstanding, the burden of TB in the country is higher than previously estimated—a prevalence rate of 290/100,000 compared to 90/100,000 as was earlier thought [16]. This effectively puts the current detection rate in the country at 24% of the estimated prevalence, far less than the World Health Organisation (WHO) case detection rate target (70%). A recent study [17] in Ghana revealed that the TB infection rates are driven partly by late diagnosis, which is exacerbated by fears of stigmatisation with high risks of transmission outside the household [18].

The recently released results of the 2014 GDHS showed that 83% of women and 89% of men had heard about TB. Seventy-eight percent of women and 81% of men correctly knew that TB is spread through the air by coughing. Despite these appreciable levels of knowledge about the disease, approximately 33% of women and 25% of men would want to keep TB in their families as a secret. This work therefore explores the characteristics of Ghanaians who believe that TB in a family should be kept a secret. This understanding is important for behavioural change interventions aimed at increasing case findings/diagnosis. Also, while there is a body of literature on the different dimensions of TB-related stigma and status disclosure, the majority of these studies have focused on patients [14, 19, 20]. As the world, including Ghana, embarks on an ambitious agenda of eliminating TB by 2030, we need more evidence to address tendencies that can impede identifying, diagnosing and treating all suspected cases. In the Ghanaian context too, the nature of housing and residential arrangements of families make the study imperative. Whereas the family may connote a group of persons consisting of parents and children living together in a household (nuclear), it could also refer to descendants of a common ancestor (extended). In Ghana, it is a norm, rather than an exception for two or more nuclear families to co-reside in the same house [21]. In such circumstances, disclosing the status of a family member with TB can be useful for reducing or cutting further transmissions in houses and the larger community. Characterizing the individual, household and community level factors associated with aversion towards TB status disclosure becomes important for public health.

## Methods

### Data

The 2014 edition of Ghana Demographic and Health Survey (GDHS) is the most recent of five surveys carried out since the inception of demographic and health surveys in 1987/88. Specific questions asked on TB were about transmission, knowledge of availability of cure and whether they (respondents) would keep it a secret when a family member gets TB. The TB module was added to the survey at the request of the National TB Control Programme, Ghana, to help the programme generate nationally representative data on aspects of TB knowledge, attitudes and practices (KAP).

The survey was implemented by the Ghana Statistical Service (GSS) in collaboration with ICF Macro and Ghana Health Service (GHS). A multi-staged sampling was used; the first stage of the sampling involved a selection of 427 clusters of which 216 were from urban areas and the remainder from rural areas (211). The selection process was based on a list of an updated enumeration areas of the 2010 Population and Housing Census. An enumeration area (EA) in Ghana typically is made up of about 180 to 200 households or 750 persons. Using a simple random technique, approximately 30 households were selected from each cluster, yielding a total of 12,831 households. Women (15–49 years) and men (15–54 years) were interviewed. In each of the households sampled, all eligible women, defined as being in the age category of 15–49 years as well as being a *de facto* resident of the household were main criteria for inclusion. In half of the 12, 831 sampled households, all eligible men between 15 and 54 years who gave informed consent were interviewed. The cut-off of age was set at these limits mainly because the survey targeted women and men of reproductive ages. At the end of the survey, 9,656 women (response rate 97%) and 4,609 (response rate 95%) were interviewed. The Ghana Health Service Ethics Review Committee and the Institutional Review Board of ICF Macro reviewed and approved the protocol for the survey. Each respondent provided written consent prior to participation.

### Dependent variable

The dependent variable for this work was derived from a question which asked survey participants whether they would “keep secret when a family member gets TB”, with three responses — “no = 0”, “yes = 1” and “don’t know = 8”. However, “No” and “Don’t Know” were grouped together [No and Don’t Know = 0 and Yes = 1] to generate a binary variable. The existing literature is not unanimous on whether “No” and “Don’t know” present the same understanding. Whereas others contend that “No” and “Don’t Know” connote the same meaning (e.g. [22]), EA Waters, JL Hay, H Orom, MT Kiviniemi and BF Drake [23] disagree. In this paper, however, the former view was applied given the small proportion of “Don’t Know” responses among both men (0.77%) and women (2%). EKM Darteh, DT Doku and K Esia-Donkoh [24] have resorted to a similar approach of treating “No” and “Don’t Know”.

### Independent variables

Following extant literature (e.g. [8, 10]) the following individual level [education (no education = 0, primary = 1, secondary = 3 & higher = 4), age (15–19 = 1, 20–24 = 2, 25–29 = 3, 30–34 = 4, 35–39 = 5, 40–44 = 6, 45–49 = 7, 50–54 = 8, 55–59 = 9), marital status (never in union = 0, currently in union/living with a man/woman = 1& formerly in union/living with a man = 2) household [wealth (poorest = 1, poorer = 2, middle/average = 3 richer = 4, richest = 5)], community level [ethnicity (Akan = 1, Ga/Dangme = 2, Ewe = 3, Mole-Dagbani = 4, Gurma = 5 & Others = 6), religion (Orthodox Christian = 1 [Catholic, Presbyterian, Methodist and Anglican], Pentecostal/Charismatic = 2, Other Christian = 3, Islam = 4, & Traditional/Spiritualist = 5), region (Western = 1, Central = 2, Greater Accra = 3, Volta = 4, Eastern = 5, Ashanti = 6, Brong-Ahafo = 7, Northern = 8, Upper East = 9 and Upper West = 10), type of place of residence (urban = 1, rural = 2)] were extracted for the analysis. Extent of exposure to the following media channels – radio, newspaper/magazine and television (not at all = 0, less than once a week = 1 & at least once a week = 2) were also considered in the regression analysis. Another variable was respondents’ views about whether HIV in the family should be kept a secret. There are some indications that people who are infected with TB but who know they are HIV-negative are reluctant in disclosing their status as well as those of family members for fear of being labelled as HIV patients [25], justifying the need to test this assumption empirically.

### Statistical analysis

Two types of statistical analysis were performed – descriptive and binary logistic regression. First, the descriptive bivariate association between independent factors aforementioned were conducted. The associated Chi-square test scores are also presented. In the next stage of the analysis, binary logistic regression models were estimated for women and men separately. Apart from the need to isolate whether men and women were affected by the same factors or not, the nature of the data generated by Measure DHS do not easily allow merging due to the possibility of conflicting unique identifiers of respondents. In each case, the variables were inputted in the regression model simultaneously as I aimed at examining the net effects of each of the explanatory variables. The complex design of the survey was factored into the analysis by applying the *svy* command in STATA (College Station, Texas 77845 USA) in all the estimations.

## Results

### Descriptive results

This section is in two parts: the first part presents brief characteristics of the respondents interviewed and the second component looks at the bivariate associations between characteristics of the respondents and their views about keeping TB of a family member secret or otherwise. The results on respondents’ characteristics were as follows: in terms of age, the highest proportion of both men and women were between the ages of 25–29 years. The data also shows that a majority of women and men had attained at least secondary education. A higher proportion of households were in the richest quintile. Urban residents were slightly more than rural residents. Relative to ethnicity and religion, there were more Akan^2^ and Pentecostals than the other groups [Data not shown].

In this section, I present the bivariate associations between the independent variables and the dependent variable [Table 1]. The results show that not keeping TB status in the family secret among men increased with age; similar patterns were noted among women (*p* < 0.001). It is again shown in Table 1 that whereas increasing levels of formal education made women more inclined not to keep TB in the family a secret; the educational influence on men was minimal. For instance, among men, more than 8 out of 10 with no education compared to about 7 out of 10 with secondary and higher education were disposed to disclose TB status of a family member. The poorest men (82%; *p* < 0.001) and the richest among women (73%; *p* < 0.001) were more open to TB status disclosure. The data also revealed significant regional differences in views about keeping TB within the family secret for both women and men: less than half of women (48.6%; *p* < 0.001) from the Upper East and just about half (50.6%; *p* < 0.001) of men in the Ashanti region responded “No” to the question on keeping TB secret. No significant urban-rural differences were found among women, albeit minimal disparities existed among men (*p* < 0.016). Religious variations were significant among women but not for men. On the other hand, ethnicity showed a strong association with keeping TB secret. A majority of formerly married men and currently married/in-union women indicated that TB of a family member would not be kept secret. Exposure to media (newspaper/magazine, radio and television) was correlated with whether TB in the family would be kept secret or not [Table 1]. Given the close association between HIV and TB, I also assessed whether views about disclosing HIV in the family was associated with intentions to disclose TB status. A very strong association was established — 49.8 and 52.8% of women and men who would keep HIV/AIDS in the family secret would do it for TB too. In sum, 68% of women and 75% of men reported they would not keep TB a secret if a member of their family became infected.

**Table 1:** Descriptive bivariate association between independent factors and intentions to keep TB status secret

Explanatory variables	Females		Males	
	N	%	N	%
Individual level				
Education	*χ* ^2^ = 25.3; *p* < 0.001		*χ* ^2^ = 17.1; *p* < 0.001	
No education	1,138	65.8	342	84.1
Primary	1,163	65.0	410	77.0
Secondary	4,681	67.1	2,532	73.5
Tertiary	576	78.0	492	77.5
Age group	*χ* ^2^ = *p* < 0.001		*χ* ^2^ = 57.2; *p* < 0.001	
15–19	1,070	57.8	633	66.9
20–24	1,275	65.8	499	68.4
25–29	1,340	67.7	527	76.6
30–34	1,161	69.9	486	77.1
35–39	1,093	69.9	425	76.3
40–44	878	72.9	413	81.9
45–49	739	69.7	319	80.9
50–54	–	–	276	78.5
55–59	–	–	196	83.1
Marital status	*χ* ^2^ *=* 15.3; *p* < 0.001		*χ* ^2^ = 32.2; *p* < 0.001	
Never married	2,415	63.6	1,525	70.1
Married/In-union	4,287	69.8	2,043	78.6
Formerly married	856	66.4	209	81.4
Household level				
Household wealth quintile	*χ* ^2^ = 34.5; *p* < 0.001		*χ* ^2^ = 6.3; *p* < 0.175	
Poorest	862	64.8	529	81.6
Poorer	1,237	64.3	658	76.7
Average	1553	65.7	725	75.5
Richer	1,879	66.6	873	71.2
Richest	2,027	72.6	991	74.6
Community variables				
Ethnicity	*χ* ^2^ = 168.0; *p* < 0.001		*χ* ^2^ = 54.7; *p* < 0.001	
Akan	4,173	65.7	1,961	71.1
Ga/Dangme	600	67.8	352	83.4
Ewe	1,014	81.1	521	84.1
Mole-Dagbani	935	60.7	484	78.3
Gurma	259	71.9	177	80.5
Others	575	64.0	281	15.7
Religion	*χ* ^2^ *=* 33.9;*p* < 0.001		*χ* ^2^ = 8.1; *p* < 0.089	
Orthodox Christian	1,852	67.3	956	75.9
Pentecostal	3,210	68.9	1,158	73.0
Other Christian	1,228	61.1	698	77.7
Moslem	995	74.6	625	79.3
Traditional/Others	272	74.6	339	79.3
Region	*χ* ^2^ = 330.0; *p* < 0.001		*χ* ^2^ = 339.7; *p* < 0.001	
Western	935	65.4	436	72.3
Central	776	56.2	379	88.7
Greater Accra	1,681	77.4	823	87.8
Volta	538	83.8	279	85.3
Eastern	658	68.9	381	76.8
Ashanti	1,582	66.6	735	50.6
Brong-Ahafo	596	52.1	302	63.2
Northern	390	69.1	264	95.3
Upper East	251	48.6	99	73.2
Upper West	151	57.8	75	69.3
Residence	*χ* ^2^ = 0.01 *p* < 0.893		*χ* ^2^ = 5.7; *p* < 0.016	
Urban	4,410	67.9	2,052	72.1
Rural	3,149	66.7	1,725	79.3
Media exposure variables				
Newspaper/Magazine	*χ* ^2^ = 6.5; *p* < 0.038		*χ* ^2^ = 13.4; *p* < 0.001	
Not at all	5,904	66.9	2,335	73.6
Less than once a week	880	66.7	721	81.8
At least once a week	769	66.4	719	81.4
Radio	*χ* ^2^ = 7.3; *p* < 0.038		*χ* ^2^ = 25.0; *p* < 0.001	
Not at all	910	67.2	166	55.3
Less than once a week	2,426	67.7	577	82.4
At least once a week	4,223	67.4	3,034	75.1
Television	*χ* ^2^ = 16.1; *p* < 0.001		*χ* ^2^ = 11.7; *p* < 0.003	
Not at all	1,430	64.9	540	73.6
Less than once a week	2,019	68.8	685	82.8
At least once a week	4,110	67.4	2,552	73.7
Views about HIV disclosure				
Keeping HIV & AIDS in family secret	*χ* ^2^ = 1.2;*p* < 0.001		*χ* ^2^ = 799.5; *p* < 0.001	
No	3,381	88.9	2,097	93.1
Yes	4,116	49.8	1,665	52.8

### Multivariable logistic regression results

Here, results from multivariable logistic regression are presented. Considered by age of respondents, women between 30–34 years (OR = 0.564, *p* < 0.001) were less motivated to keep TB of a family member secret referenced to those 15–19 years. For men, it was those in older ages — 55–59 years (OR = 0.350, *p* < 0.001) who were unlikely to keep TB in the family secret. Women who had attained tertiary education (OR = 0.582, *p* < 0.001) had more positive attitudes towards disclosing TB within a family. Persons who would keep HIV and AIDS secret would also keep TB secret; closely 7 times higher (OR = 6.992, *p* < 0.001) among women and almost 10 times higher (OR = 9.870, *p* < 0.001) among men [Table 2] as against those who indicated willingness to disclose HIV and AIDS.

**Table 2:** Multivariable binary logistic regression results on factors associated with keeping TB secret in Ghana

Predictor variables	Females		Males	
	OR	95% CI	OR	95% CI
Individual level variables				
Education (No education)				
Primary	1.049	[0.870,1.264]	0.940	[0.594,1.487]
Secondary	0.851	[0.715,1.013]	0.920	[0.635,1.332]
Tertiary	0.582^***^	[0.433,0.782]	0.863	[0.536,1.389]
Age group (15–19)				
20–24	0.701^**^	[0.564,0.871]	0.648^**^	[0.470,0.893]
25–29	0.713^**^	[0.568,0.895]	0.545^**^	[0.377,0.787]
30–34	0.564^***^	[0.442,0.719]	0.465^***^	[0.309,0.701]
35–39	0.576^***^	[0.449,0.738]	0.558^*^	[0.352,0.886]
40–44	0.632^***^	[0.493,0.811]	0.473^**^	[0.294,0.760]
45–49	0.801^*^	[0.669,0.960]	0.498^**^	[0.310,0.801]
50–54	-	-	0.602^*^	[0.371,0.978]
55–59	-	-	0.353^***^	[0.201,0.622]
Marital status (Never married)				
Married/In-union	1.010	[0.856,1.191]	1.046	[0.761,1.438]
Formerly married	1.131	[0.896,1.427]	0.937	[0.586,1.496]
Household factor				
Household wealth quintile (Poorest)				
Poorer	1.046	[0.841,1.300]	0.784	[0.560,1.096]
Average	1.070	[0.838,1.366]	0.752	[0.507,1.116]
Richer	0.923	[0.704,1.209]	0.817	[0.534,1.252]
Richest	0.855	[0.630,1.161]	0.793	[0.485,1.296]
Community-related variables				
Ethnicity (Akan)				
Ga/Dangme	1.431^*^	[1.080,1.897]	0.904	[0.603,1.353]
Ewe	0.805	[0.630,1.029]	0.951	[0.636,1.424]
Mole-Dagbani	1.091	[0.847,1.406]	0.877	[0.601,1.281]
Gurma	0.872	[0.591,1.287]	1.145	[0.649,2.021]
Others	1.105	[0.860,1.419]	1.305	[0.876,1.943]
Religion (Orthodox Christian)				
Pentecostal	0.919	[0.793,1.066]	1.143	[0.892,1.464]
Other Christian	0.898	[0.739,1.091]	0.903	[0.685,1.191]
Moslem	0.976	[0.795,1.199]	0.841	[0.603,1.171]
Traditional/Others	0.821	[0.600,1.124]	1.133	[0.784,1.637]
Region (Western)				
Central	1.231	[0.834,1.816]	0.457^**^	[0.277,0.755]
Greater Accra	0.605^**^	[0.415,0.883]	0.423^***^	[0.265,0.675]
Volta	0.551^**^	[0.355,0.855]	0.624	[0.349,1.116]
Eastern	0.730	[0.496,1.074]	0.765	[0.526,1.114]
Ashanti	0.957	[0.664,1.378]	1.845^*^	[1.117,3.049]
Brong-Ahafo	1.276	[0.897,1.816]	1.397	[0.890,2.191]
Northern	1.004	[0.627,1.606]	0.206^***^	[0.0998,0.427]
Upper East	1.299	[0.832,2.028]	1.198	[0.684,2.097]
Upper West	0.806	[0.520,1.248]	1.110	[0.648,1.902]
Residence (Urban)				
Rural	0.858	[0.702,1.048]	0.803	[0.597,1.079]
Media exposure				
Newspaper/Magazine (Not at all)				
Less than once a week	1.064	[0.860,1.318]	0.685^**^	[0.517,0.908]
At least once a week	1.008	[0.796,1.276]	0.811	[0.619,1.063]
Radio (Not at all)				
Less than once a week	0.922	[0.764,1.111]	0.797	[0.483,1.316]
At least once a week	1.048	[0.873,1.257]	0.657	[0.416,1.038]
Television (Not at all)				
Less than once a week	0.732^**^	[0.603,0.888]	0.960	[0.683,1.349]
At least once a week	0.952	[0.794,1.141]	1.229	[0.893,1.691]
Views about HIV/AIDS status disclosure				
Disclosure of HIV & AIDS in family				
No	1	[1, 1]	1	[1, 1]
Yes	6.992^***^	[6.026,8.113]	9.870^***^	[7.894,12.34]
N	7,492		3,762	
AIC	259.8		3204.1	

^*^ *p* < 0.05, ^**^ *p* < 0.01, ^***^ *p* < 0.001

Reference category in parenthesis

Regionally, women in the Volta Region (OR = 0.551, *p* < 0.01) and men in the Northern Region (OR = 0.206, *p* < 0.001) compared to respondents from the Western region were less likely to want to keep TB in the family secret. On the other hand, men in the Ashanti (OR = 1.845, *p* < 0.05) and Brong-Ahafo, Upper East and Upper West regions were more likely than those in the Western region to express aversion to disclosing TB status of a family member. In contrast, it was women in the Brong-Ahafo, Northern and Upper East regions that were more likely than those in the Western region to oppose disclosing TB in the family.

## Discussion

An unwillingness to disclose a particular disease condition maybe influenced by the possibility of stigma and discrimination. Nevertheless, for an infectious disease like TB, which is transmitted through the air (coughing and sneezing), disclosure becomes important. This analysis therefore examined factors associated with keeping TB as a secret or otherwise among a cross-sections of Ghanaians between the ages of 15–59 years.

Women, unlike men, expressed more aversion towards disclosing TB in the family. The proportions reported in this paper for men (75%) and women (68%) are similar to the proportions [women: 67% and men: 77%] in a previous study [5]. Evidence suggests that the hesitation of women in disclosing their own TB infections or those of their relatives may partly emanate from concerns about being stigmatized which threatens social relations [8, 26]. The stigma can lead to interruptions in social relations such as marriage [6]. Underlying this are limited mobility and financial dependency among women, leading to fears of public disclosure of themselves and those in their families.

As seen in this paper, the issue of low mobility and dependency of women can be noted in the views expressed by women with highest level of education. Education is one of the important indicators of women’s empowerment (e.g. [27]). Women in this category are therefore in a better position to know about the availability of cure for TB, the mechanisms involved in transmission, either through formal school learning or exposure to public education messages. Such wealth of information may have contributed to be their positive attitudes towards disclosure. Similar effect of higher education was found on men; although not significant. However, the importance of highlighting more on women here is the disproportionate burden of TB stigma on women compared to men.

A finding worth highlighting is the highly significant relationship between attitudes towards HIV/AIDS disclosure in the family and TB. There is no question about the close clinical impact of HIV on activation of latent TB and the co-infection of the two has led to what V Bond and L Nyblade [25] described as double stigma. Despite the emerging double stigma of both diseases, HIV seems to evoke higher levels of stigma than TB as initial public representations of the disease connoted death [28, 29]. TB-related stigma is also linked to HIV in some studies [8]. Not surprisingly, those who expressed willingness to disclose HIV in the family were willing to do the same for TB.

The study also revealed varying regional differences in attitudes towards disclosing TB status in the family. The exact reasons for this observation maybe difficult to perceive, especially so on the account of inconsistencies between women and men even in the same region. However, we tend to side with earlier cultural epidemiologists who note that spatial variations in perceptions about specific diseases emanate from long held community opinions about those diseases. A Khandoker, M Khan, A Krämer and M Mori [30] observed spatial differences in perceptions about TB transmission, awareness of cure and attitudes towards disclosure. For instance, in the Volta region, the *Ewe* language, which is the main language renders TB as *yomokpe*, which means death or tomb. The physical deterioration of untreated TB principally accounts for this local description. Perhaps, such community beliefs about the disease may be accounting for the observed regional disparities.

The study also revealed a linear trend with age in terms of positive attitudes towards disclosure in favour of older respondents. The age disparities in attitudes towards disclosure could be related to generational gaps in knowledge and practices about TB. Thus, older people might have the benefit of knowing how TB and its management has evolved between the past and the present. In fact, a study with old and young people in some Ghanaian communities have revealed more positive perceptions of older people about TB compared to younger people. While older people thought that the context of living with TB now was better than in the past, young people expressed fear and gloom for one to be infected with TB [31].

This paper is not without limitations. The dependent variable on which this analysis was based was measured hypothetically. To that extent, the responses provided may vary when the reality of TB infection struck in the family. Coupled with this is the fact that the analysis was based on cross-sectional data and this calls for caution in making categorical inferences. Notwithstanding this, the representativeness and the quality of the DHS datasets provides important insights into the existing situation with regards to attitudes of Ghanaians about keeping TB as a family secret.

## Conclusion

The results point to reasonable amount of individual, household and community level differences in attitudes towards disclosure. The unwillingness towards TB disclosure may partly be driven by fear of stigmatisation and discrimination. Addressing TB-related stigma and discrimination can enhance positive attitudes towards TB disclosure. For an infectious disease such as TB, openness towards disclosure is important for preventive public health.

## Abbreviations

AIDS: Acquired immune deficiency syndrome
GHS: Ghana Health Service
GSS: Ghana Statistical Service
HIV: Human immunodeficiency virus
KAP: Knowledge, attitudes and practices

## Footnotes

1 Enablers support is a kind of incentives provided to TB patients in the course of treatment. These may include call vouchers, food and transportation allowance to facilitate the treatment process. These incentives are intended to reduce patient default particularly among the very poor who may default treatment due to financial difficulties.

2 Akans are one of the ethnic groups in Ghana; they constitute about 47% of Ghana’s population

## References

[1] Ortblad KF, Lozano R, Murray CJ (2013). An alternative estimation of tuberculosis incidence from 1980 to 2010: methods from the global burden of disease 2010. Lancet.

[2] World Health Organisation (2015). Global tuberculosis report 2014.

[3] Yuen CM, Amanullah F, Dharmadhikari A, Nardell EA, Seddon JA, Vasilyeva I, Zhao Y, Keshavjee S, Becerra MC (2015). Turning off the tap: stopping tuberculosis transmission through active case-finding and prompt effective treatment. Lancet.

[4] Courtwright A, Turner AN (2010). Tuberculosis and stigmatization: pathways and interventions. Public Health Rep.

[5] Amo-Adjei J. Policy and social context for tuberculosis control in Ghana. PhD Thesis, Department of Population and Health, University of Cape Coast; 2013.

[6] Johansson E, Long N, Diwan V, Winkvist A (2000). Gender and tuberculosis control: perspectives on health seeking behaviour among men and women in Vietnam. Health Policy.

[7] Daftary A (2012). HIV and tuberculosis: the construction and management of double stigma. Soc Sci Med.

[8] Somma D, Thomas B, Karim F, Kemp J, Arias N, Auer C, Gosoniu G, Abouihia A, Weiss M (2008). Gender and socio-cultural determinants of TB-related stigma in Bangladesh, India, Malawi and Colombia. Int J Tuberc Lung Dis.

[9] Goffman E. Stigma: Notes on the management of spoiled identity. London: Simon and Schuster; 2009.

[10] Karim F, Chowdhury A, Islam A, Weiss MG (2007). Stigma, gender, and their impact on patients with tuberculosis in rural Bangladesh. Anthropol Med.

[11] Dodor EA, Kelly S (2009). ‘We are afraid of them’: attitudes and behaviours of community members towards tuberculosis in Ghana and implications for TB control efforts. Psychol Health Med.

[12] Van Rie A, Sengupta S, Pungrassami P, Balthip Q, Choonuan S, Kasetjaroen Y, Strauss RP, Chongsuvivatwong V (2008). Measuring stigma associated with tuberculosis and HIV/AIDS in southern Thailand: exploratory and confirmatory factor analyses of two new scales. Trop Med Int Health.

[13] Rwemisisi J, Wolff B, Coutinho A, Grosskurth H, Whitworth J (2008). ‘What if they ask how I got it?’Dilemmas of disclosing parental HIV status and testing children for HIV in Uganda. Health Policy Plan.

[14] Zolowere D, Manda K, Panulo B, Muula A, Panulo DZKMB, Muula JA (2008). Experiences of self-disclosure among tuberculosis patients in rural Southern Malawi. Rural Remote Health.

[15] Amo-Adjei J, Awusabo-Asare K: Reflections on tuberculosis diagnosis and treatment outcomes in Ghana. Arch Public Health. 2013;71(22). doi:10.1186/2049-3258-71-22. 10.1186/2049-3258-71-22 PMC3765431 23971675

[16] National Tuberculosis Control Programme (2015). National TB prevalence and associated risk factors in Ghana In.

[17] Osei E, Akweongo P, Binka F (2015). Factors associated with DELAY in diagnosis among tuberculosis patients in Hohoe Municipality, Ghana. BMC Public Health.

[18] Verver S, Warren RM, Munch Z, Richardson M, van der Spuy GD, Borgdorff MW, Behr MA, Beyers N, van Helden PD (2004). Proportion of tuberculosis transmission that takes place in households in a high-incidence area. Lancet.

[19] Dodor EA, Kelly S, Neal K (2009). Health professionals as stigmatisers of tuberculosis: insights from community members and patients with TB in an urban district in Ghana. Psychol Health Med.

[20] Dhingra V, Khan S (2010). A sociological study on stigma among TB patients in Delhi.

[21] Addai-Sundiata JH. Family dynamics and residential arrangements in Ghana. The Changing Family in Ghana. Proceedings of National Research Conference, Ghana, 25th-27th January 1996. Ghana Universities Press, Accra. pp. 64–85.

[22] Groothuis PA, Whitehead JC (2002). Does don’t know mean no? Analysis of’don’t know’responses in dichotomous choice contingent valuation questions. Appl Econ.

[23] Waters EA, Hay JL, Orom H, Kiviniemi MT, Drake BF (2013). “Don’t know” responses to risk perception measures implications for underserved populations. Med Decis Making.

[24] Darteh EKM, Doku DT, Esia-Donkoh K (2014). Reproductive health decision making among Ghanaian women. Reprod Health.

[25] Bond V, Nyblade L (2006). The importance of addressing the unfolding TB‐HIV stigma in high HIV prevalence settings. J Community Appl Soc Psychol.

[26] Johansson E, Long N, Diwan V, Winkvist A (1999). Attitudes to compliance with tuberculosis treatment among women and men in Vietnam. Int J Tuberc Lung Dis.

[27] Mahmud S, Shah NM, Becker S (2012). Measurement of women’s empowerment in rural Bangladesh. World Dev.

[28] Alonzo AA, Reynolds NR (1995). Stigma, HIV and AIDS: An exploration and elaboration of a stigma trajectory. Soc Sci Med.

[29] Herek GM, Glunt EK. An epidemic of stigma: Public reactions to AIDS. American Psychol Association. 1988;43(11):886–91. 10.1037//0003-066x.43.11.886 3063145

[30] Khandoker A, Khan M, Krämer A, Mori M (2011). Knowledge about tuberculosis transmission among ever-married women in Bangladesh. Int J Tuberc Lung Dis.

[31] Bonsu F, Awusabo-Asare K, Amo-Adjei J (2015). A report on national survey of knowledge, attitudes and practices about tuberculosis in Ghana.

